# An Address, Introductory to the Third Course of Lectures in the Atlanta Medical College

**Published:** 1857-06

**Authors:** J. Boring

**Affiliations:** Professor of Obstetrics, &c., Atlanta, Georgia


ATLANTA

Medical and Surgical Journal.

Vol. II.] JUNE, 1857. [No. 10

### ORIGINAL COMMUNICATIONS.

### ARTICLE I.

*An Address Introductory to the Third Course of Lectures in the Atlanta Medical College.* By J. Boring, M. D., Professor of Obstetrics, &c., Atlanta, Georgia.

Gentlemen of the Class:

My colleagues have, in the absence of the appointee, devolved upon me the pleasing duty of tendering you, in their names, and those of the good people of this City, a hearty welcome to the Halls of the "Atlanta Medical College."

Happily, to this hour, no misunderstandings, no heartburnings have arisen between the Students of this College and citizens of the place.

All have alike rejoiced in the almost unparallelled success of the Institution, and mutually striven for the perpetuity of fraternal relations and sentiments.

Those who constituted our first and second classes, returned to their homes, bearing with them the confidence, the kindliest feelings and respect, of the whole population of Atlanta, whilst in return, *Atlanta* has not ceased to feel that they are her honored sons—those in whose welfare she is deeply interested.

And, I may be permitted to add, that nothing has transpired thus far, by which the utmost friendship and cordiality of intercourse, between Teachers and Students, have been in the slightest degree interrupted.

To us, these are subjects of pleasant review, whilst at the same time, they inspire the hope that such will mark our future history.

We cherish the fond hope that *one* Medical College at least, will mature to the full stature, and attain to the gravity and dignity of age, free from the unhappy prejudices which so generally attend these institutions.

That you, gentlemen, heartily respond to this sentiment, we doubt not, and in return, we pledge for ourselves and the people, that reciprocity which we have a right to expect.

On the part of the Faculty, allow me in this connection to say, that all we have, and are, are yours. We promise you that nothing promotive of the objects of your attendance here, shall be wanting. For the four months to come, we are yours.

It is cause of deep regret, with both you and ourselves, that we are denied the privilege of the expected Introductory on the present occasion.

The Professor of Surgery was, as you are aware, appointed to this honor, but from an unexpected delay in Europe, found it impracticable to meet the engagement.

While you, gentlemen, feel the *disappointment*, it is *mine* to suffer both the disappointment and embarrassing responsibilities devolved.

Under the stress of circumstances, I have hurriedly cast about me for some appropriate theme, some subject, in the discussion of which, I might hope both to please and instruct.

The custom of "Introductories" has so long and extensively prevailed, as to have brought under contribution, almost every subject of importance to the Profession, and rendered it next to impossible to present anything new. It has, however, occurred to me, that *one* subject, belonging to Medicine, has failed to receive the attention which its importance demands, and that I cannot render a better service, for the present, than that of its presentation. It is

THE USE AND ABUSE OF TOBACCO.

That the subject announced comes fully within the range of Medical investigation is not questioned, and hence without an apology for its introduction, I shall proceed to its investigation.

It will not be inappropriate, and it is hoped, not uninstructive, before noticing the medicinal, and other properties of

Tobacco, to glance briefly at its history, especially since, as is well known, it exercises, well-nigh, universal dominion over the appetites and habits of mankind.

It is, perhaps, generally understood that this singular plant, was first discovered among the natives of Tropical America, and thence was introduced into Europe.

That Europeans saw it first in this Country, and thence transferred it to Portugal, Spain and France, is rendered quite certain; but that it was cultivated in Asia, and possibly other portions of the Earth, long anterior to the discovery of the American Continent, can hardly be doubted.

Humbolt says, "The Tobacco plant has been cultivated from time immemorial, by the natives of Oronoko. It does not appear, however, to have been known to Europeans prior to the discovery of America; though it is not improbable that the Asiatics were acquainted with it long before that time, as Palas, Rumphius, and Laureiro, have supposed. But it is not probable, I think, that the Europeans learned the use of it from the Asiatics, as Ulloa has endeavored to show."—*Humbolt's Personal Narrat ve, vol.* 5, *p.* 666.

On the arrival of Columbus, at Cuba, in 1492, he sent a small company of his men in search of a large Province, which was reported by the natives to abound in gold, and as being in the interior of the Island. Disappointed in the object of their search, they were returning to the ship, when they saw the natives going about with firebrands in their hands, and certain dried herbs, which they rolled up in a leaf, and lighting one end, put the other into their mouths, and continued inhaling and puffing out the smoke. A roll of this kind they called tobacco, a name, since transferred to the weed itself." —*Washington Irvings Life and Voyages of Columbus—Abridged Edition, p.* 69.

From this country, it was sent by Hernandes, de'Toledo, into Spain, and Portugal, and thence to France, by Nicot, in 1559–60.

"In 1756, on the return of Sir Francis Drake, with the Colonists from Virginia, the practice of smoking was introduced into England, and being adopted by Sir Walter Raleigh, and other courtiers, soon become common." *Pereiras' Materia Medica and Therapeutics—vol.* 2, *p.* 332.

Its cultivation and use, except for medicinal purposes, were strongly opposed in England, and in fact throughout the enlightened world.

Books were written against it, and laws prohibiting its use were enacted, but all to no purpose. It spread, as if by the power of magic, over the world, and now wields a more universal sway over mankind than any single article of food or luxury known to commerce.

In the eloquent language of another, "The history of Tobacco forms a curious item in the annals of our race."

Next to intoxicating liquors, there is no substance which has gained such an ascendancy over human taste and appetite as Tobacco. There is no nation on the face of the globe, civilized or savage, where it has not found its way.

Europe, Asia, Africa and America, all are familiar with it. There is no condition of society in which it is not a favorite guest.

You find it in the palace and in the poor-house—in the stately mansion and the humble cottage—in the work-shop and the billiard room; the lonely exile solaces his weary hours with it—the joyous freeman exults in its influence. Philosophy muses under its power.

Poetry is inspired, and hardy labor cheered by tobacco.

Wherever man is found, its influence is felt and acknowledged. The citizen whiffs his perfumed cigar—the poor man smokes his sooty pipe—the sailor chews his delicious quid—the matron rejoices in her pinch of snuff.

On the mountain top and in the lonely valley—on the land and on the broad expanse of ocean—in the dark mines of Pennsylvania, and in the glittering halls of Paris—on the rugged hills of Switzerland, and in the gold bearing valleys of California—amid the snows of the North, and under the burning suns of the Tropics—in battle and peace—in storm and in calm—in wealth and in poverty—in health and in sickness—the king and the subject—the master and the slave—youth, manhood and old age—all, all love the magic power of tobacco."—*Beck.*

This unexampled triumph over mankind, is the more remarkable from the fact, that the uneducated palate abhors the article. Nothing, mineral or vegetable, is so disgusting, so nauseous and overwhelmingly prostrating. So strong are the impressions of a first or second trial, in this upward march to manhood, that no incentives are sufficient to induce its repetition until time has obtunded the recollection of its loathing sickness and death-like sweats.

Perhaps, however, the most singular fact, developed in this investigation, is, that of all classes, the learned Professions are the most consecrated devotees to the use of tobacco. *The Clergy*, the *Legal* and *Medical professions are its greatest consumers.*

Having briefly noticed the history of tobacco, it is pertinent to enquire next into its use.

And here the question naturally arises, "Is there any use for it?" The inquiry is not, whether it has done most good or harm, but, has it an appropriate place in the list of blessings bestowed upon man? Were I called upon to say whether the world would have been the better without the knowledge of it, I should unhesitatingly answer in the affirmative. This however, has arisen, not from the article itself, but its *abuse* by mankind. It *has* its place, and *that* place is in the Materia Medica. Here, and here only, God intended the knowledge and application of its extraordinary powers.

It is a potent remedy, a virulent poison, producing effects, analogous to those of Digitalis, Veratrum, and Prussic Acid, and should therefore never be given, except by the physician or surgeon, and by them, only in cases of unquestionable necessity. It is a narcotic, sedative, emetic, diuretic, expectorant, etc.—*See works on Mat. Med.*

The analyses of Tobacco have detected among its active principles: "*Nicotin*"; an empyreumatic, volatile oil, and "*Nicotianin*," an alkaline substance, all of which are deadly poisons.

Every part of the plant contains these principles, and readily imparts them through almost any conceivable medium.

The fumes, as seen from the pipe and cigar, are largely impregnated with empyreumatic oil, the deposites from which, are exceedingly poisonous. It is said that the Hotentots are aware of this fact, and often use the matter contained in their pipe stems to destroy serpents. Mr. Barrow, witnessed an instance in which this substance was applied to the tongue of a poisonous snake; the reptile stretched itself out, became stiff, and died in an instant.—*Cyclopedia of Practical Medicine.*

I may mention, in this connexion, that instances have occurred, under my observation, in which the juice of Tobacco, spit on the heads or into the mouths of serpents, was followed by similar effects.

On the subject of the "Modus Operandi" of this article, little need be said here. Experiments render it highly probable that its action is directed both to the nervous and circulatory systems, affecting the functions of the brain and heart, to a serious, and even fatal extent, especially, when administered in large doses. But, whatever may be its mode of action, or the obscurity by which it is invested, *one thing* is demonstrable, and that is, that it is an agent of tremendous power, and requires in its administration the utmost skill and precaution of the Medical mind.

As a remedial Agent, Tobacco is seldom employed by the Profession, and is chiefly valuable in cases demanding a powerfully relaxing medicine. Tetanus, Intussusception, Hernia, etc., have been successfully treated under its influence.

It is administered in the form of Decoction, Ointment, and the application of the wet leaves over the region of the Stomach, its effects being readily obtained by any method of exhibition.

Having ascertained the *use* of Tobacco, let us next inquire into the subject of its *abuse.*

Before proceeding with this branch of the discussion, it may not be amiss to explain what is here intended by the use of the term "*abuse.*" I mean perversion—misapplication; a wrong appropriation of this, or any other article. Its application to purposes other than those intended by the Great Author of all good.

That every such perversion is an *abuse*, and must in the very nature of things, work mischief, will not be denied. The only question here to be settled is, whether Tobacco, was intended as an article of food or luxury. That it is Medicinal has been seen. That it is nutritious, has, I believe, never been assumed, except by the disgusting Tobacco-Worm, and the Musky Goat. Man, though the largest consumer, never conceives of his quid or cigar, as contributing to his nourishment. All *he* claims for it is, the gratification of an unnatural appetite.

Physiologically, it is impossible that a substance possessing narcotic and sedative qualities in so high a degree, should act other than as an *exhauster*, and in proportion to the extent of its application, destroy the vital energies. So true is this, and so generally understood, of Tobacco, that it is everywhere used and recommended for the prevention and reduction of corpulency.

But, the position assumed by the advocates of the quid and cigar is, that "it is a Luxury." This has generally been conceded by those who have opposed its use, and its condemnation has been sought alone, in what are held to be its *direct evils*. Now this concession is wrong. It is founded in error. Tobacco *is not a luxury*. It is a Medicine, a poison, a destroyer of the normal powers and functions of the animal economy, and thus produces a state of the nervous system, in which a *morbid* pleasurable sensation is experienced, but not properly luxurious.

It is a fact of universal experience and observation, that the human palate, in its normal state, loathes and detests the article; the brain becomes dizzy, the heart grows faint and convulsive, and the whole man quails under its potent influence.

The appetite for Tobacco, is a creature of its own morbid action, the gratification of which, is of necessity morbid also, and therefore utterly incompatible with all just ideas of luxury. A diseased action, the result of a diseased, or vitiating cause, cannot be held to be a luxury. The very proposition is an absurdity. As well may the Inebriate talk of the "Luxury" of being drunk, or the Debauchee, of the "Luxury" of his hallucinations in a fit of "delirium tremens." It is diseased action—nervous derangement, and in fact, strictly speaking, *not a state of life*.

That it has the effect, when habitually used, and the nervous system has become the victim of its power, to soothe and exhilarate, is not here denied; but that *such* an effect is incompatible with *sanity*, and is therefore *not* luxurious, seems to me absolutely certain.

Waiving this view of the subject for the present, let us enquire, whether such exhilaration as that claimed for Tobacco, and on account of which, it is justified as a luxury, is compatible with the physiological laws of man's economy, and may be safely protracted. That all such excitement is morbid, has been seen, and if morbid, violative of the laws of health, seems inevitable. Let it not be said in reply, that its action is sought, as a remedy, in certain cases, and cannot therefore be morbid. We bleed, and blister, and vomit our patients when sick—we stimulate and we depress the heart and arteries, but no man is so profoundly blind, as to hold that these are *therefore*, in harmony with the healthy or sound state of the patient—they are for the use of the diseased, in the removal of his malady. Who ever thinks of perpetuating these remedies and their effects, for the safety of his patient? And why not? They cured, as has Tobacco, and if its remedial qualities, ascertained in the treatment of disease, constitute its medicinal excitement essential to, or compatible with the laws of health, why not of the others? If, in this case, we plead for the perpetuity of morbid excitement, and its compatibility with the physiological laws of our economy, why not in the cases of analagous remedies, such as alcohol, opium and kindred articles? Where is the intrinsic difference?

Again. If such effects in perpetuity, are in harmony with the physiological laws of our economy, why is it, that in all cases, the exhilaration, *thus* produced, is followed, not only by a corresponding, but *far surpassing* depression, and that the agent employed, must be had in constantly increasing quantities, to keep up the *degree* of excitement? Is it not obvious that the action is morbid—in violation of the laws of health, and just in proportion to its obtunding effects upon the vital energies, the *power* of the exciting cause must be increased to obtain an equal response?

The undeniable physiological truth is, that such agents are productive of *undue action* on the part of all those organs coming within their range of influence, which must inevitably result in their impairment.

But, I shall be told of those who have used Tobacco from early youth to old age, and have developed no evil results. The fact assumed, I doubt, but allowing its correctness for the present, I may point to the drunkard of half a century, in proof that habitual drunkenness is also compatible with health. He has been a drunkard *fifty years*, and although his whole man, *soul*, *body* and *spirit*, are *steeped* in alcohol, his iron constitution seems unimpaired. But while he has *lived* a drunkard, who can tell how many, with far less indulgence, *have found a drunkard's grave?* They, perhaps, died unconscious of the fact, that they were slain by alcohol, but *their* ignorance on the subject, operates no mitigation of the startling truth. The habit of drinking intoxicating liquors, often superinduces a state of general bad health, which in the end, developes dyspepsia, paralysis, apoplexy, or some form of chronic disease, by which life is destroyed, the unhappy victim all the while supposing himself to be sinking into the grave, according "to the course of nature."

Startling as the proposition may be, there can be no doubt, that hundreds and thousands of *sober* men, so called, and so believing of themselves, die of alcohol, and though never suspected by others or themselves, fill a drunkard's grave.

So of Tobacco. Multitudes are slain by its insidious invasions, in relation to whom, such a thing never was suspected. They were not directly, and at the instant, poisoned to a fatal extent, but slowly invaded, until the very citadel of life was invested by the insidious influence—the final blow was struck.

That the *habit* of using Tobacco, seems in some sort to educate the system to its influence, is no proof that mischief is not being done. Death may not, probably *will* not *immediately* result from the article itself, but the deadly impressions are going on, and the final results will be fatal. The perpetual dripping of water will wear away the hardest granite. Its impressions may be so slow as to elude the most scrutinizing observations, but at last the *huge rock is swept away.*

The young man may give no visible signs of the innovations upon his health, by this poison, but an early grave, or premature old age, with tremulousness, paralysis of body and mind, and general inervation will finally demonstrate its *fatal potency.*

But Tobacco is an *active*, a *virulent poison*, and therefore positively contra indicated as an article of luxury.

This proposition is sustained by every authority of the Profession.

Wood and Bache, in the United States Dispensatory, say of one of its active principles, "Nicotina," "In its action on the animal system, it is one of the most virulent poisons known.

A *drop* of it in the state of concentrated solution was sufficient to destroy a dog; and small birds perished at the approach of a tube containing it."—[*See article Tobacco, U. S. Dispensatory.*]

Professor Dunglison states that, "in large doses, Tobacco is one of the most violent acro-narcotic poisons. When given in the form of decoction, or applied to abraded surfaces it has caused death." *Dunglison's Materia Medica and Therapeutics, vol.* 1, *p.* 135.

*Pereira*, in describing its effects, says, "The more prominent symptoms are, nausea, vomiting, and in some cases, purging, with extreme weakness and relaxation of the muscles, depression of the vascular system, (manifested by feeble pulse, pale face, cold sweats and tendency to faint,) convulsive movements, followed by paralysis and a kind of torpor, terminating in death. [*Periera's Materia Medica, vol.* 2, *p.* 327.]

"In large doses," says Professor Beck, "it is a virulent poison, acting principly upon the brain and heart. It impairs the action of the heart, causing a sense of fluttering, excessive faintness, copious perspiration, sense of alarm, sickness and vomiting, coldness of the skin, feebleness of pulse, convulsions and death. *Beck's Mat. Med.*, *p.* 340.

The following strong language on this subject, is published in the "Cyclopedia of Practical Medicine," vol. 4, p. 150. "Tobacco is another sedative of great power. The experiments of Sir B. Brodie have rendered it probable that there are two efficient principles in Tobacco: one an empyreumatic, volatile oil, which operates directly on the brain and nerves of sensation, or on the sensibility of the system; the other, a saline substance (nicotiana) which appears to influence, chiefly the motor nerves, confining its sphere of action particularly to the heart, which it renders insensible to its natural stimulus, the blood, and thereby causing death.

In whatever manner this volatile oil is procured, its effects are so powerful on the animal economy, that when it is applied to an abraded surface, or introduced into the system, it causes almost instant death.

Numerous instances are recorded in medical works, in which Tobacco has produced fatal results, by being applied to the scalp, over the region of the stomach, to abraded surfaces, and in the form of decoction, both in the hands of medical men and under the practice of the domestic circle. So frequent are these occurrences, as to have excited a just apprehension in the mind of every intelligent writer on the subject, and hence the cautions with which their works abound, as to its administration.

I saw an account but a few days since, published in a Texas newspaper, in which it was stated that a little girl, ten or twelve years of age, had come to her death by the use of snuff, in the disgusting practice of "*dipping.*"

How strong the infatuation, which adopts as a *luxury*, a poison so deadly and uniform in its effects!

It is not only *poisonous*, but is one of the most virulent, acro-narcotic poisons with which the medical mind is acquainted, standing in this respect, *pre-eminent* in its class.

The melanchollly effects of Tobacco, are however, by no means confined to the physical man. As should be expected, from its action on the brain and nervous system, the stomach and process of digestion, and the circulation; seriously affecting the heart and arteries, the intellectual faculties suffer the most disastrous consequences. A moments reflection must satisfy the observing mind, that any cause, the effects of which, so seriously modify the action of these vital organs and functions, will inevitably involve the mental powers. How can the mind continue healthy, and perform its office, when all the organs on which it depends, or with which it is in connexion are diseased? A corrupt fountain cannot send forth a pure stream. *A diseased brain cannot nourish and sustain a sound mind.* As soon may we expect fever without increased action of the heart and arteries. In accordance with these views, it may here be stated, as a well established fact, that *Lunacy* is a frequent result of this habit. The reports of Lunatic Asylums, in the United States, have within the few last years, demonstrated the truth of this statement. Of the unhappy victims to this, heaviest of calamities, an astounding proportion is from the professions, especially the christian ministry.

There are at this moment, several ministers of the Gospel, *demented*, and driven from their high and holy avocations, to confinement in Asylums for the insane, in this country, by the habitual use of tobacco.

A short time since, I saw published in a widely circulating and respectable religious newspaper, an account of the Rev. ——, Pastor of the —— church, in Virginia, who cut his throat in a fit of insanity, and the attending physicians attributed his insanity to the excessive use of this article. What an end for a Christian minister!

It is a nice question, whether, or how far *self-murder* may be extenuated by insanity, when *that* insanity is the result of a voluntary and needless indulgence. Is it less excusable for a man to kill himself with alcohol or a knife, than with tobacco? Where is the difference? You say, such was not the *intention* in the case of using tobacco. Neither is it the intention of the drunkard or debauchee.

I cannot consent to dismiss this subject without remarking, that the human mind is not only subject, as the body, to diseased action, but in proportion to its superiority of delicateness and consequent impressibility, is more easily disordered, and incapitated. I do not mean to say, that all degrees of mental disease, amount to *insanity*, as this term is generally understood and used; but, I *do* mean to say that *any* degree of such diseased action *is derangement*, and liable to *progress to a fatal consummation.*

There can be no doubt, but that multitudes are the subjects of mental disease, and consequently bereft of the normal action of this high endowment, without its being known to themselves, or suspected by their most intimate friends.

If this be true, and who can doubt it, is it a matter of surprise, that those who are *perpetually* under the influence of an acro-narcotic poison, the direct action of which, is upon the brain and nervous system, should end their melancholy course in *total insanity?* Is it not rather, just what we should expect?

Such then, are *some* of the physiological views of this subject. It is not contended or believed, that tobacco, will in all cases produce the effects described above; nor is it desired to create an extravagant apprehension of its evils: this would be unfortunate to my purpose. That these, however, are its legitimate fruits, and that they are realized in an incomparably greater proportion of instances than the masses of mankind are in the habit of believing, I entertain not the shadow of a doubt, and that no man has a *right* to expect less for himself in the indulgence of the habit.

Aside from these considerations, there are conclusive reasons for the disuse of tobacco.

On a different occasion, I should feel bound to present the moral and religious view of this subject.

*That the expenditure of money, the waste of time, the damage of health, the beclouding the mind, the obtunding the moral sensibilities and powers, and the destruction of life, have resulted from the habit, and may again accrue, will not be doubted; and that a cause of voluntary action, producing, or even liable to produce such results, involves responsibilities of *stupendous* magnitude, is to my mind, beyond all question.

It requires no labored argument, to show that, although the habit of using Tobacco does not always lead to the drinking saloon and gambling table, there is a *tendency* to reciprocity, and, with multitudes, the one leads to the other. There is something of affinity, by which they seem bound in association.

*Personally*, the habit is one of great inconvenience, and inevitable polution.

There are no limits to its demands, and no circumstances, by which its victim can be, even temporarily, released from its manacles.

In the family and social circle—on the highway and in the study—in the Court-House, and in the Temple of God—in the presence of ladies, and in the room of the sick—everywhere and always, its claims are imperious. And then, the

* The annexed table, showing the annual production, cost and consumption of Tobacco in the United States, is based upon the best information that can be had of those engaged in its manufacture and sale. It is not claimed that the estimates are nicely accurate, but that they sufficiently approximate the facts, for all valuable purposes:

| | |
|---|---|
| Annual production, | 400,000,000 lbs. |
| Value, at 12 cents per lb., (low,) | $48,000,000 |
| Amount manufactured and chewed in the U. S. annually, | 60,000,000 lbs. |
| Cost, say 30 cents per lb., | $18,000,000 |
| Amount smoked in pipes and cigars, much of which is imported at high prices, | 15,000 000 |
| Snuff used, | 8,000 000 |
| Total cost of consumption, | $41,000,000 |

I entertain no doubt that these estimates are all below the actual figures in the case

What a sum of money to be annually expended by enlightened, Christian Americans, in the gratification of a morbid appetite, and the destruction of health and life! A sum, sufficient to send Missionaries to every tribe of man under the heavens, and to kindle the fires of salvation upon the altar of every benighted soul of man.

discolored lips, the foul teeth, the offensive breath, the stained goatee and bosom, to say nothing of the disgusting pool of juice so often found at the feet of the devotee, are sufficient to create an utter abhorrence of the practice.

And now, gentlemen, what shall be said of the physician, who so far forgets the elevated character of his Profession, as to allow himself ushered into the room, and seated at the bedside of a lady patient, his very person an offence to refinement and taste, and his breath, a sickening stench in her nostrils?

Is it not an outrage of unmitigated turpitude upon the sex, and shame upon our humane profession?

But I shall be told that ladies also use Tobacco, and hence men should be excused.

It is true, ladies use Tobacco, and that too, in the most disgusting manner; but who is willing to justify his own act by a practice so foul, and so universally condemned by every man of sense and taste?

Except one of her sex, rioting in alcoholic fumes, and blundering into the ditch, it is hard to conceive of a more disgusting object, than that of a woman "*dipping.*"

Think of it. A woman! a bottle of Mackaboy! a filthy stick—stained lips—yellow teeth—polluted breath—sick headache—nervous irritability—novel-reading—sleepless nights—hysterical spasms—blue devils, and hob-goblins! and she a *wife*, a *mother!*

Gentlemen, if I desired to wither your prospects for all time to come, and to embitter the cup of life, I would ask that such a woman should be yours.

As an expression of my best wishes, for your happiness and success, I pray that you may escape this "untoward generation."

A few days since, when on board a car with an unusual number of passengers, I saw a young girl of fourteen or fifteen years of age, indulging in this, foulest of habits. The stick, (brush) used on the occasion was absolutely black, had evidently done good service, and although the operation of "dipping," rubbing, sucking and spitting, was disgusting beyond endurance, this hopeful adept, seemed to become almost ethereal under the Narcotic, and finally stretched herself on the seat, seemingly, insensible to the burning shame of her degrading habit and position. What a spectacle! who can plead for it? Let the voice of the profession be raised against it, and let *example* enforce the teaching.

